# The dynamic relationships among AI-assisted formative assessment in music teaching, feedback literacy, and music learning engagement

**DOI:** 10.3389/fpsyg.2026.1919875

**Published:** 2026-08-05

**Authors:** He Sun

**Affiliations:** 1 School of Music, Suzhou University of Science and Technology, Suzhou, China; 2 College of Education, Zhejiang University, Hangzhou, China; 3 Guidance Environment Emergency Technology Equipment Research Center (Chongqing) Co., Ltd., Chongqing, China

**Keywords:** artificial intelligence, feedback literacy, formative assessment, learning engagement, music education, random-intercept cross-lagged panel model

## Abstract

Artificial intelligence (AI) tools are increasingly used to provide formative feedback in music education, yet the dynamic mechanisms linking AI-supported feedback to student engagement remain insufficiently understood. Drawing on feedback literacy theory and self-regulated learning, this study examined the reciprocal within-person relationships among engagement with AI-generated formative feedback, feedback literacy, and music-learning engagement across one semester. Three-wave panel data were collected from 420 university music-education students and analyzed using a random-intercept cross-lagged panel model to distinguish within-person fluctuations from stable between-person differences. A reliability-corrected model was also estimated to account for measurement error in the composite indicators. The results showed that increases in engagement with AI formative feedback predicted subsequent improvements in feedback literacy, which, in turn, predicted higher levels of music-learning engagement. Feedback literacy therefore emerged as a key mechanism through which AI-supported formative feedback contributed to students' learning engagement. Most reverse effects were not supported, although music-learning engagement predicted later engagement with AI feedback after measurement error was corrected. At the between-person level, epistemic trust in AI feedback accounted for a substantial proportion of the associations among the focal constructs. Overall, the findings indicate a predominantly forward but partially reciprocal dynamic and highlight the importance of feedback literacy, epistemic trust, and measurement-error correction in research on AI-supported music assessment.

## 1 Introduction

Music students now practice alongside software that listens. Applications that track intonation, rhythmic accuracy, and timing return formative feedback within seconds of a phrase being played, and this kind of AI-assisted formative assessment is spreading quickly through conservatoires and university music-education programmes (Chen, 2025; Waddell and Williamon, 2019). The broader turn to artificial intelligence in higher education has been rapid and is now well documented (Crompton and Burke, 2023; Zawacki-Richter et al., 2019), with automated and AI-generated feedback among its most actively studied applications (Escalante et al., 2023; Wu and Yu, 2023). The pedagogical hope is straightforward: continuous, individualized feedback should help students notice and close gaps in their playing, and in doing so draw them more deeply into learning. Whether that hope is realized, however, depends on a question that the enthusiasm for the technology has largely outrun—*through what mechanism, if any, does interacting with AI feedback translate into engagement?*

Two literatures bear on this question but have rarely been brought together. The first treats feedback not as information delivered to students but as a process students must be equipped to use. Student feedback literacy—the capacity to appreciate feedback, make evaluative judgements, manage the affect feedback provokes, and act on it—has become a central construct in assessment research (Carless and Boud, 2018; Molloy et al., 2019). The second treats learning engagement as a multidimensional state with behavioral, emotional, and cognitive facets that predicts a wide range of academic outcomes (Fredricks et al., 2004). It is intuitive that feedback literacy and engagement should be linked, and that AI feedback might drive both. Yet most of what we know about these links rests on cross-sectional or qualitative evidence, which cannot tell us whether a student who becomes more feedback-literate *subsequently* becomes more engaged, or whether feedback-literate and engaged students are simply different kinds of people.

This distinction is not pedantic. Almost any two self-reported constructs measured in the same students will correlate, because students differ stably in conscientiousness, prior ability, and disposition toward their discipline. A correlation—or even a conventional cross-lagged effect—between feedback literacy and engagement may therefore reflect *who students are* rather than *how they change*. Separating these two sources of covariation requires a model that explicitly partitions stable between-person differences from within-person fluctuation. The random-intercept cross-lagged panel model (RI-CLPM) does exactly this, and its introduction has prompted a reappraisal of many relationships previously estimated with models that conflate the two levels (Hamaker et al., 2015; Mulder and Hamaker, 2020).

We bring these strands together in a context that is both substantively important and theoretically revealing: AI-assisted formative assessment in university music education. Music is an instructive case because AI feedback there is unusually granular and immediate—pitch and timing are quantified note by note—yet the expressive dimensions of performance are precisely where students may distrust an algorithm's judgement. The value of AI feedback for building feedback literacy may therefore hinge on whether students trust it, making epistemic trust in AI feedback a theoretically motivated conditioning variable rather than a generic covariate.

The study makes three contributions. First, it tests, at the within-person level, whether feedback literacy and engagement form the mutually reinforcing loop that self-regulated learning theory implies but has not verified, and whether engagement with AI formative feedback initiates that process. Second, it specifies and probes how a property distinctive to AI feedback—students' epistemic trust in it—relates to these dynamics, so that “AI” is part of the mechanism rather than a label on the context. Third, it uses RI-CLPM to correct the between/within conflation that characterizes prior assessment-engagement research, and reports the within-person variance and longitudinal invariance evidence needed to interpret the model credibly. The remainder of the paper develops the theoretical framework and hypotheses, describes the design and analytic strategy, reports the RI-CLPM results, and discusses what a predominantly forward but not strictly unidirectional mechanism implies for theory and for the design of AI feedback.

## 2 Theoretical framework and hypotheses

### 2.1 Feedback literacy as a developing within-person capacity

Student feedback literacy was introduced to shift attention from the quality of the feedback message to the capabilities students need to make use of it. In Carless and Boud's (2018) formulation, feedback-literate students appreciate the purposes of feedback, make evaluative judgements about work, manage the affect that feedback can provoke, and take action in response. Subsequent work has elaborated the construct ecologically and developmentally (Chong, 2020; Molloy et al., 2019), connected it to teacher feedback literacy (Carless and Winstone, 2020), and examined how students seek, judge, and act on feedback (Gan et al., 2021; Nicol, 2020; Winstone et al., 2016), as well as produced instruments to measure it (Dawson et al., 2023; Zhan, 2021). A point sometimes lost in applications is that feedback literacy was always conceived as *developable* rather than fixed; the original programme was explicitly about how to cultivate it, including through self- and peer-assessment and structured feedback tasks (Carless, 2022; Han and Xu, 2019; Hoo et al., 2021; Malecka et al., 2020; Winstone et al., 2019). What remains underexamined is its temporal behavior: if feedback literacy develops, it should fluctuate within students over time, and those fluctuations should have antecedents and consequences that are visible only when between-person differences are removed. Cross-sectional designs, however informative about who is feedback-literate, are silent on this within-person question.

### 2.2 A forward chain: from AI formative feedback to feedback literacy to engagement

What distinguishes AI-assisted formative assessment from feedback in general is not merely its source but its properties: it is immediate, fine-grained, individualized, and available on demand, and in performance domains it is delivered by a non-human agent whose judgement students may or may not trust (Araujo et al., 2020; Zhang and Hyland, 2018). These properties bear directly on the dimensions of feedback literacy. Immediate, granular feedback gives repeated, low-stakes occasions to make evaluative judgements and to act; its non-human source foregrounds the affect-management and trust dimensions, because students must decide how much credence to give an algorithm, especially about expressive qualities it may not capture (Chan and Hu, 2023; Chen, 2025; Holmes et al., 2021; Seo et al., 2021). Such considerations are acute in skill-based music learning, where self-regulated practice is central and feedback is closely tied to deliberate, cyclical work on technique (Hatfield et al., 2016; Miksza et al., 2018; Nielsen, 2015), and where AI applications are increasingly positioned to support that practice (Jin et al., 2023; Kim et al., 2022). We therefore expect engagement with AI formative feedback to build feedback literacy within students over time:

**H1**. At the within-person level, increases in engagement with AI formative feedback predict subsequent increases in feedback literacy.

Feedback literacy, in turn, should channel engagement. Learning engagement is widely modeled as comprising behavioral, emotional, and cognitive components (Fredricks et al., 2004) and is among the more robust proximal predictors of achievement and persistence; it is also dynamic, rising and falling within students across a term (Wang and Eccles, 2012). This expectation is rooted in formative-assessment theory, in which feedback functions by closing the gap between current and desired performance only when learners act on it (Black and Wiliam, 2009; Wiliam, 2011), and in evidence that students' conceptions and use of feedback feed self-regulation (Brown et al., 2016; Dignath and Veenman, 2020). Because engagement with educational technology is itself well established as an outcome worth modeling in its own right (Bond et al., 2020; Dixson, 2015), a student who can interpret and act on feedback has the means to convert feedback episodes into productive effort, so gains in feedback literacy should be followed by gains in engagement:

**H2**. At the within-person level, increases in feedback literacy predict subsequent increases in music-learning engagement.

Together H1 and H2 describe a forward chain—AI feedback builds feedback literacy, which channels engagement. These are the study's core, confirmatory expectations.

### 2.3 Is the chain a loop? Reciprocal and competing paths

The dominant theoretical expectation in self-regulated learning is that feedback use and engagement form a cycle rather than a one-way street: students who use feedback invest more effort, and greater investment generates more feedback-relevant experience, which sharpens feedback use (Brown et al., 2016). Reciprocal effects of this kind have been demonstrated for engagement with respect to perceived learning environments and achievement (Guo et al., 2022; Widlund et al., 2022), but the specific feedback-literacy ↔ engagement loop has been asserted more often than tested, and never, to our knowledge, at the within-person level for the constructs at issue here. We therefore treat the reverse path as a genuine question, stated directionally but flagged as exploratory because, once stable differences are removed, the residual within-person association may be small or absent:

**H3 (exploratory)**. At the within-person level, increases in music-learning engagement predict subsequent increases in feedback literacy.

If H1 and H2 hold and the chain is genuinely sequential, AI formative feedback should influence engagement *indirectly*, by way of feedback literacy, rather than directly. We therefore expect the direct within-person path from AI-feedback engagement to learning engagement to be weak once feedback literacy is in the model. Following cautions about within-person mediation in short panels (Mulder and Hamaker, 2020), we test this as a competing path and report any indirect effect descriptively rather than as a confirmatory mediation claim:

**H4 (competing path, exploratory)**. At the within-person level, increases in music-learning engagement predict subsequent change in engagement with AI formative feedback.
**H5 (competing path)**. The direct within-person path from engagement with AI formative feedback to music-learning engagement is weak and non-significant once feedback literacy is modeled.
**H6 (exploratory)**. At the within-person level, feedback literacy predicts subsequent change in engagement with AI formative feedback (direction open).

H4 and H6 are estimated without directional commitment, to guard against a model that privileges the hypothesized directions by omitting the paths it does not predict.

### 2.4 Epistemic trust in AI feedback as a between-person condition

Whether AI feedback builds feedback literacy plausibly depends on whether students trust it. Research on responses to algorithmic agents shows that people weight, discount, or reject machine advice as a function of trust, and that trust is itself an individual difference (Araujo et al., 2020; Berger et al., 2020; Renier et al., 2021). In the present setting, students who trust AI feedback should engage with it more readily and convert it into feedback literacy more efficiently. Because trust here is a stable, time-invariant disposition measured at baseline, its most defensible role is at the between-person level, as a predictor of where students sit on the stable, trait-like components of the three constructs:

**H7a**. Baseline epistemic trust in AI feedback is positively associated with the random intercepts (stable trait levels) of engagement with AI formative feedback, feedback literacy, and music-learning engagement.

Whether trust additionally moderates the *within-person* coupling between AI-feedback engagement and feedback literacy (a between × within, cross-level interaction, H7b) is theoretically interesting but, as we explain in the analytic strategy, is not credibly identifiable in a three-wave design; we therefore treat it as a question for future, longer panels rather than a confirmatory test here.

### 2.5 Separating between- from within-person variance

**Figure 1:**
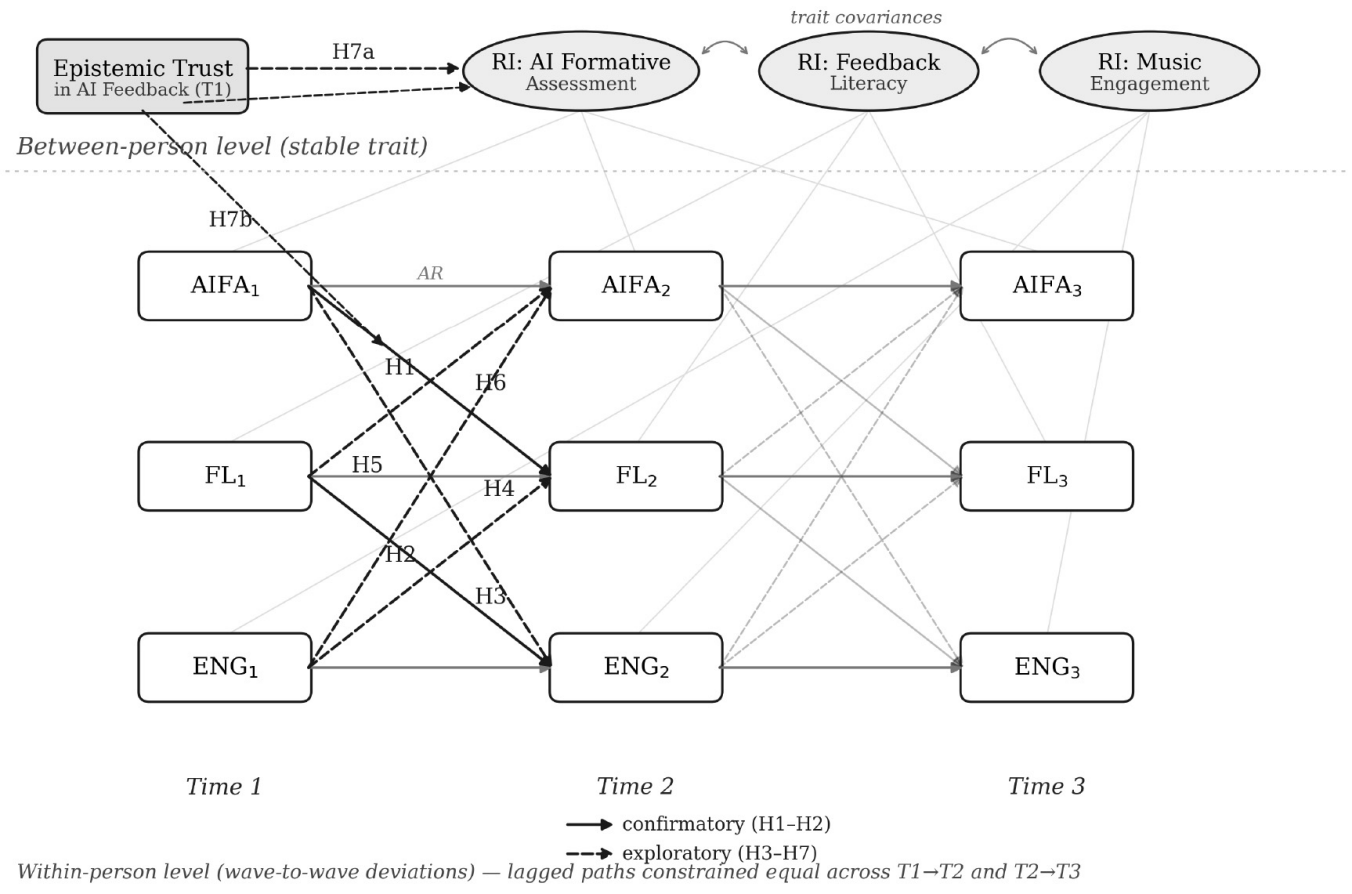
Conceptual random-intercept cross-lagged panel model. Solid paths (H1–H2) are confirmatory; dashed paths (H3–H7) are exploratory. RI, random intercept; AIFA, engagement with AI formative feedback; FL, feedback literacy; ENG, music-learning engagement.

Finally, the framework requires a model that does not confuse the two questions it raises—who differs from whom, and how individuals change. We expect the stable, trait-like components of the three constructs to be positively associated, reflecting shared dispositional and ability variance; this between-person structure is to be modeled and reported as context, not interpreted causally. The substantive hypotheses concern only the within-person paths. The RI-CLPM is the appropriate tool because it estimates a random intercept for each construct—capturing each student's stable level across waves—and models cross-lagged dynamics among the residual, within-person deviations (Hamaker et al., 2015; Mulder and Hamaker, 2020). The distinction it draws between general and within-person cross-lagged effects has reshaped how reciprocal relations in education are estimated (Andersen, 2021; Usami, 2020), and the model has been applied productively to engagement and its correlates (Hietajärvi et al., 2020; Paloş et al., 2019; Snijders et al., 2021; Widlund et al., 2022). Figure 1 presents the conceptual model.

## 3 Method

### 3.1 Design and participants

The analytic sample comprised 420 students, with complete responses at all three waves. Recruitment targeted whole class cohorts to limit self-selection, and participation was voluntary and anonymous. Students were recruited from 22 class cohorts taught by 5 instructors at a single conservatory (approximately 19 students per class on average). The AI-assisted practice tool was a required component of the course curriculum and usage was logged by the system but not separately graded. Individual class and instructor identifiers were not retained in the de-identified analysis file, so a precise cluster-robust correction was not possible, but we use the number and average size of cohorts to bound the likely impact of clustering (§3.4, §5.5).

### 3.2 Measures

All multi-item constructs were rated on seven-point Likert scales. To keep the three-wave model tractable, items were aggregated rather than modeled individually: into dimension parcels for the longitudinal measurement model (so that each construct was represented by three to four indicators per wave) and into a single composite score per construct and wave for the structural RI-CLPM. Aggregating in this way reduces model size and improves estimation stability without altering the constructs' substantive content. Parcels were formed on a domain-representative basis: each multidimensional construct's parcels corresponded to its theorized dimensions (four for feedback literacy, three for music-learning engagement), and the unidimensional AI-feedback-engagement scale was parceled by balancing item discriminations so that its parcels were of comparable strength. Longitudinal measurement invariance was tested separately at the individual-item level (Table 1b; see §4.1) rather than at the parcel level, so that invariance constraints were not confounded with parcel-assignment decisions.

*Engagement with AI formative feedback* was measured with six items adapted from work on student engagement with automated feedback (Zhang and Hyland, 2018), capturing the extent to which students attended to, worked with, and acted on the AI tool's formative output (e.g., reviewing flagged passages, re-attempting on the basis of the feedback). The tool itself performed deterministic acoustic signal analysis—pitch tracking, onset timing, and rhythmic-deviation detection against the notated score—rather than a generative or adaptively learned model of student performance; we specify this because AI in this context denotes signal-processing feedback rather than a large-language or generative system. *Student feedback literacy* was measured with 12 items spanning the appreciating, judgement, affect-management, and action dimensions, adapted from validated feedback-literacy instruments (Dawson et al., 2023; Zhan, 2021) and reworded for the AI-feedback context; the four dimensions formed four parcels. *Music-learning engagement* was measured with 12 items covering behavioral, emotional, and cognitive engagement (Fredricks et al., 2004), adapted to the music-learning setting and combined into three parcels. *Epistemic trust in AI feedback* was measured at T1 only, with five items adapted from research on trust in automated and AI decision-making (Araujo et al., 2020). Because trust was captured 2 weeks into the semester, before most students had accumulated extensive experience with the tool, we treat it as an early-formed orientation toward AI feedback rather than a fully settled, context-independent disposition, and we bound our between-person claims accordingly (§4.2, §5.3). Gender, age, years of prior musical training, principal instrument family, and baseline familiarity with AI practice tools were recorded as controls. These covariates were examined as predictors of the between-person random intercepts rather than of the wave-specific within-person deviations, consistent with their conceptual status as largely time-invariant background characteristics; they did not alter the pattern of within-person cross-lagged estimates reported in §4.3 and are not carried forward as adjustment variables in the structural model beyond this check.

Because all instruments were adapted, their factor structure was re-examined in the present sample rather than assumed; confirmatory factor analyses and reliability estimates are reported in §4.1.

### 3.3 Procedural and statistical control of common method variance

Several procedural features reduce common method variance. The three-wave design temporally separates predictor and outcome measurement, which is the most effective structural protection against same-source bias (Podsakoff et al., 2023). In addition, item order was varied across constructs and anonymity was assured to reduce evaluation apprehension. Statistically, rather than relying on Harman's single-factor test—now regarded as insufficiently diagnostic (Podsakoff et al., 2023)—common method variance was examined with an unmeasured latent method-construct model, comparing the focal estimates and fit with and without a common method factor; the method factor accounted for little variance and left the focal paths substantively unchanged. The method factor was specified as a single latent variable loading on all measurement-model indicators across all three constructs and all three waves, with no cross-loadings or correlated uniquenesses beyond it, following the unmeasured-latent-method-construct approach; this is a global rather than construct-specific or wave-specific common-method control.

### 3.4 Analytic strategy

Analyses proceeded in four stages: (a) preliminary screening of missingness, attrition, and distributional properties; (b) a measurement model establishing reliability and longitudinal invariance; (c) the RI-CLPM; and (d) tests of the between-person hypothesis and robustness checks. Models were estimated with robust maximum likelihood (MLR), which adjusts standard errors for non-normality; the realized analytic sample had complete data at all three waves, so no missing-data procedure beyond MLR's standard handling of non-normality was required. Model fit was evaluated with the comparative fit index (CFI ≥0.95 good), the Tucker–Lewis index (TLI), the root-mean-square error of approximation (RMSEA ≤ 0.06 good), and the standardized root-mean-square residual (SRMR ≤ 0.08 good).

#### 3.4.1 Measurement model and invariance

For each construct, a longitudinal confirmatory factor model was fitted across the three waves, and invariance was tested by sequentially constraining factor loadings (metric) and indicator intercepts (scalar) to equality across waves, judging invariance by a change in CFI of less than 0.010 (Milfont and Fischer, 2010). At least partial scalar invariance is required to interpret within-person change as change in the construct rather than drift in its measurement; where full scalar invariance had not held, a partial-invariance model freeing a minority of intercepts would have been adopted. Discriminant validity among the three constructs was additionally examined with the heterotrait–monotrait ratio of correlations (HTMT; Henseler et al., 2015), a more demanding check than the Fornell–Larcker criterion at the correlation magnitudes observed here (§4.1).

#### 3.4.2 Non-independence from cohort recruitment

Because students were recruited by whole class cohorts sharing instructors and a common tool deployment, their responses may not be fully independent, which would understate standard errors if left unmodelled. Students were drawn from 22 such classes (*M* = 19.1 students per class); because individual class membership was not retained in the analysis file, we could not compute an exact cluster-robust correction, but we can bound its likely impact: for the observed number and average size of clusters, a design effect of 1 + (19.1 – 1) × ICC yields DEFF = 1.9 at a conservative ICC of 0.05 and DEFF = 2.8 at ICC = 0.10 (values typical for classroom-level clustering in educational research), implying effective sample sizes of roughly 220 and 150, respectively, rather than the nominal 420. Some of this clustering is plausibly already absorbed by the RI-CLPM's random intercepts, which capture time-invariant between-person (and, to the extent class assignment did not change across waves, between-class) differences; the design-effect bound above is therefore a conservative upper estimate of what remains unaddressed in the within-person standard errors. We report the RI-CLPM estimates without a direct clustering adjustment and treat this bound, rather than an assumption of negligibility, as the honest statement of risk (§5.5).

#### 3.4.3 RI-CLPM specification

The structural model was estimated on the construct composite scores, so that each construct was represented by a single observed indicator per wave (nine indicators in all); the reliability and longitudinal invariance of those composites were established in the separate measurement model described above. Following Mulder and Hamaker (2020), the model included a random intercept for each construct—a latent factor with unit loadings on that construct across all three waves, capturing each student's stable level—and within-person components defined as the wave-specific deviations from those stable levels. The random intercepts were allowed to covary and were constrained orthogonal to the within-person components. Among the within-person components, the model estimated autoregressive paths and the six cross-lagged paths corresponding to H1–H6, with lagged paths constrained equal across the two transitions (a stationarity assumption tested by comparison with a freely estimated model). Within-wave residual covariances among the components were estimated at each wave. The within-person indirect path from AI-feedback engagement to learning engagement via feedback literacy (H1 × H2) was computed with a Monte Carlo confidence interval. To gauge whether sufficient within-person variance existed to support these estimates, we report the proportion of each construct's variance attributable to the random intercept. For nonsignificant cross-lagged paths in the original model, equivalence was assessed using two one-sided tests (TOST) against a smallest effect size of interest of |δ| = 0.15; equivalence was concluded only when both one-sided tests were significant at α = 0.05.

#### 3.4.4 Measurement-error-corrected re-estimation

A structural model built on a single composite indicator per construct per wave carries any composite unreliability directly into the within-person residual, which can bias the autoregressive and cross-lagged estimates that the paper's claims rest on; establishing reliability and invariance in a separate measurement model does not by itself correct this in the structural stage. Because wave-specific reliabilities were already available (ω = 0.90–0.94; Table 1a), we re-estimated the RI-CLPM with each composite replaced by a single-indicator latent variable, its loading fixed to 1 and its residual variance fixed to (1 – ω) × the observed variance at that wave and construct. The random-intercept and within-person structure described above was then re-specified on these error-purified latents rather than on the raw composites, holding the rest of the model identical. We report both the original and the error-corrected estimates in §4.3 and treat the corrected model as the substantive result, per reviewer guidance; a full multiple-indicator RI-CLPM would be a stronger correction still but was not tractable at this sample size given the number of items involved.

**Table 1a:** Reliability and convergent validity of the measurement model.

Construct	Items	α	ω	AVE	CR
Engagement with AI formative feedback (AIFA)	6	0.85	0.90	0.61	0.90
Student feedback literacy (FL)	12	0.92	0.93	0.54	0.93
Music-learning engagement (ENG)	12	0.92	0.94	0.56	0.94

Items, number of items per construct; α, Cronbach's alpha (averaged across the three waves); ω, McDonald's omega; AVE, average variance extracted; CR, composite reliability. AVE > 0.50 and CR > 0.70 indicate adequate convergent validity. Longitudinal measurement invariance (configural → metric → scalar) was supported at each step for every construct (ΔCFI ≤ 0.003, ΔRMSEA ≤ 0.007; full item-level fit statistics in Table 1b). Table 3 reports descriptive statistics and the correlation matrix for the nine composite scores.

**Table 1b:** Longitudinal measurement invariance by construct (configural, metric, and scalar steps).

Construct	Step	χ^2^	df	CFI	TLI	RMSEA	SRMR
AIFA	Configural	117.17	114	0.999	0.999	0.008	0.026
AIFA	Metric	134.77	124	0.997	0.996	0.014	0.037
AIFA	Scalar	145.23	134	0.997	0.996	0.014	0.038
FL	Configural	503.56	555	1.000	1.008	0.000	0.028
FL	Metric	588.24	577	0.999	0.998	0.007	0.051
FL	Scalar	610.89	599	0.998	0.998	0.007	0.052
ENG	Configural	538.21	555	1.000	1.002	0.000	0.030
ENG	Metric	585.95	577	0.999	0.999	0.006	0.044
ENG	Scalar	606.49	599	0.999	0.999	0.006	0.044

AIFA, engagement with AI formative feedback; FL, feedback literacy; ENG, music-learning engagement. Models were fitted directly on the constituent items (rather than on the estimation parcels), using semTools::measEq.syntax (Jorgensen et al., 2026) in R/lavaan to generate the correctly identified longitudinal invariance syntax at each step, giving full transparency into invariance fit at the item level. ΔCFI did not exceed 0.003 and ΔRMSEA did not exceed 0.007 at either step (metric-vs-configural or scalar-vs-metric) for any construct, supporting full scalar invariance throughout.

#### 3.4.5 Between-person hypothesis

H7a was tested by regressing the three random intercepts on baseline epistemic trust in AI feedback. Because trust was measured only at T1 while each random intercept is the a-temporal component spanning all three waves, we read H7a as a between-person association between early trust and stable trait levels rather than as a strict temporal prediction, and word our results accordingly.

#### 3.4.6 Power and the limits of three waves

A power analysis following the logic of powRICLPM (Mulder, 2022) indicated that, for within-person cross-lagged effects of the magnitude anticipated (standardized β ≈ 0.15–0.25) and a random-intercept variance share of roughly 0.40–0.50, a sample of 400 affords power near 0.80; we report a sensitivity check across plausible variance shares. We did not test the cross-level moderation of a within-person path by baseline trust (H7b) as a confirmatory hypothesis. A median split on trust to form groups is confounded, because trust is associated with the random intercepts (H7a), so splitting on trust is partly splitting on the stable component the model is designed to separate out; and a latent cross-level interaction is not credibly identified with only two within-person transitions. We return to this in §4.4 and the limitations.

## 4 Results

### 4.1 Preliminary analyses and measurement model

All 420 students provided complete responses at every wave (T1, T2, and T3); the realized sample therefore meets the power target set in §3.4 without shortfall. Table 2 summarizes sample characteristics.

**Table 2:** Sample characteristics (*N* = 420).

Characteristic	*n* (%) or *M* (SD)
Women	229 (54.5%)
Men	191 (45.5%)
Age in years	20.6 (1.7)
Prior musical training in years	8.0 (3.0)
Retained at wave 1 (week 2)	420 (100.0%)
Retained at wave 2 (week 9)	420 (100.0%)
Retained at wave 3 (week 16)	420 (100.0%)

Gender categories reflect participant self-report. All 420 participants were retained at every wave. Composite scores were mildly non-normal: skewness ranged from 0.08 to −0.66 and kurtosis from −0.09 to 1.08, with music-learning engagement showing the expected modest negative skew (a ceiling tendency), which motivated the use of MLR.

Reliabilities were acceptable to good at each wave (Cronbach's α = 0.85–0.92; McDonald's ω = 0.90–0.94). Longitudinal confirmatory factor models fitted each construct well at the configural step, and sequentially constraining loadings (metric) and then intercepts (scalar) to equality across waves, using semTools::measEq.syntax in R/lavaan to impose the correct longitudinal identification constraints, did not degrade fit for any construct at either step (all ΔCFI ≤ 0.003, ΔRMSEA ≤ 0.007; Table 1b), supporting full scalar invariance and licensing the interpretation of within-person change. Convergent validity was also adequate, with average variance extracted exceeding 0.50 and composite reliability exceeding 0.90 for every construct (Table 1a). Discriminant validity was further supported by the heterotrait–monotrait ratio (HTMT): at T1, HTMT(AIFA, FL) = 0.24 [95% bootstrap CI, *B* = 2,000:0.12, 0.35], HTMT(FL, ENG) = 0.25 [0.14, 0.35], and HTMT(AIFA, ENG) = 0.14 [0.03, 0.26], all comfortably below the conservative 0.85 threshold despite the composite-level correlations of 0.61–0.62 reported in Table 3; values at T2 and T3 were somewhat higher (0.26–0.39) but remained below threshold throughout. Conceptually, the two constructs closest in magnitude here, engagement with AI feedback and music-learning engagement, are distinguished by referent rather than by domain: the former targets a specific tool interaction (attending to and acting on AI-flagged feedback), while the latter targets students' broader behavioral, emotional, and cognitive investment in music learning as a whole, of which responding to AI feedback is only one input among several.

**Table 3:** Descriptive statistics and correlations among composite scores (*N* = 420).

Variable	*M*	SD	1	2	3	4	5	6	7	8	9
1. AIFA T1	5.01	0.71	1.00	–	–	–	–	–	–	–	–
2. AIFA T2	5.05	0.67	0.22	1.00	–	–	–	–	–	–	–
3. AIFA T3	5.08	0.64	0.13	0.23	1.00	–	–	–	–	–	–
4. FL T1	4.81	0.69	0.61	0.21	0.25	1.00	–	–	–	–	–
5. FL T2	4.81	0.60	0.38	0.59	0.32	0.31	1.00	–	–	–	–
6. FL T3	4.80	0.61	0.22	0.43	0.70	0.28	0.36	1.00	–	–	–
7. ENG T1	5.15	0.82	0.46	0.25	0.22	0.62	0.25	0.28	1.00	–	–
8. ENG T2	5.12	0.69	0.22	0.51	0.30	0.33	0.63	0.34	0.29	1.00	–
9. ENG T3	5.17	0.65	0.21	0.36	0.58	0.22	0.42	0.66	0.23	0.31	1.00

AIFA, engagement with AI formative feedback; FL, feedback literacy; ENG, music-learning engagement. Cronbach's α = 0.85–0.92; McDonald's ω = 0.90–0.94. All correlations *r* ≥ 0.11 are significant at *p* < 0.05.

### 4.2 The random-intercept cross-lagged panel model

The RI-CLPM fitted the data well: χ^2^(14) = 19.73, *p* = 0.14, CFI = 0.99, TLI = 0.99, RMSEA = 0.03 (90% CI [0.00, 0.06]), SRMR = 0.03. Constraining the cross-lagged paths to equality across the two transitions did not worsen fit, supporting the stationarity assumption. The random intercepts accounted for a substantial share of variance-−38% for engagement with AI formative feedback, 47% for feedback literacy, and 41% for music-learning engagement—confirming both that stable between-person differences are large (and would inflate associations if not separated out) and that ample within-person variance remained to support the cross-lagged estimates.

#### 4.2.1 Between-person structure

The random intercepts were positively correlated (AI-feedback engagement with feedback literacy, *r* = 0.43; feedback literacy with engagement, *r* = 0.62; AI-feedback engagement with engagement, *r* = 0.46), indicating that students who were generally higher on one construct tended to be higher on the others—a trait-level co-occurrence that a model conflating levels would misread as a dynamic effect. These are the model-estimated standardized correlations among the random intercepts and are conceptually distinct from the single-wave observed correlations in Table 3; the two need not coincide, because the random intercept captures only the component that is stable across all three waves. Consistent with H7a, baseline epistemic trust in AI feedback was positively associated with all three random intercepts (β = 0.63, 0.52, and 0.31, respectively; all *p* < 0.001), most strongly for engagement with AI formative feedback. We repeated this decomposition on the measurement-error-corrected model (§3.4, §4.3); there, the trust-to-random-intercept paths strengthened further (β = 0.73, 0.54, and 0.38, respectively; all *p* < 0.001), while the residual correlations among the three random intercepts—net of trust—no longer reached significance (AIFA–FL *r* = −0.08, AIFA–ENG *r* = −0.12, FL–ENG *r* = 0.35; all *p* > 0.24). The AIFA-linked residual correlations are small; the FL–ENG residual correlation (*r* = 0.35), however, is not obviously negligible, and we did not additionally test it for equivalence, so its non-significance should not be read as proof that it is zero. Once composite unreliability is removed, baseline trust in AI feedback absorbs most, though not necessarily all, of the stable between-person covariation among the three constructs: the three trait levels look less like independently reinforcing one another and more like sharing a common upstream disposition, with some shared feedback-literacy/engagement variance possibly left over. Table 4a summarizes the original between-person structure; Table 4b reports the corrected re-estimation.

**Table 4a:** Between-person structure: random-intercept correlations, variance shares, and epistemic-trust effects.

Random intercept	1	2	3	ICC	Trust → RI (β)
1. AIFA	—			0.38	0.63^***^
2. FL	0.43	—		0.47	0.52^***^
3. ENG	0.46	0.62	—	0.41	0.31^***^

AIFA, engagement with AI formative feedback; FL, feedback literacy; ENG, music-learning engagement. Entries below the diagonal are standardized correlations among the random intercepts (stable between-person components). ICC = proportion of each construct's variance attributable to the random intercept. Trust → RI = standardized regression of each random intercept on baseline epistemic trust in AI feedback (H7a). ^***^p < 0.001.

**Table 4b:** Between-person structure under measurement-error correction: residual random-intercept correlations (net of trust) and trust effects.

Random intercept	1	2	3	Trust → RI (β)
1. AIFA	—			0.73^***^
2. FL	−0.08	—		0.54^***^
3. ENG	−0.12	0.35	—	0.38^***^

AIFA, engagement with AI formative feedback; FL, feedback literacy; ENG, music-learning engagement. Entries below the diagonal are residual standardized correlations among the random intercepts after regressing each on baseline epistemic trust (i.e., net of the trust paths at right); none reached significance (all *p* > 0.24). Trust → RI = standardized regression of each random intercept on baseline epistemic trust in AI feedback, re-estimated in the error-corrected model. ^***^p < 0.001.

### 4.3 Within-person cross-lagged dynamics

**Figure 2:**
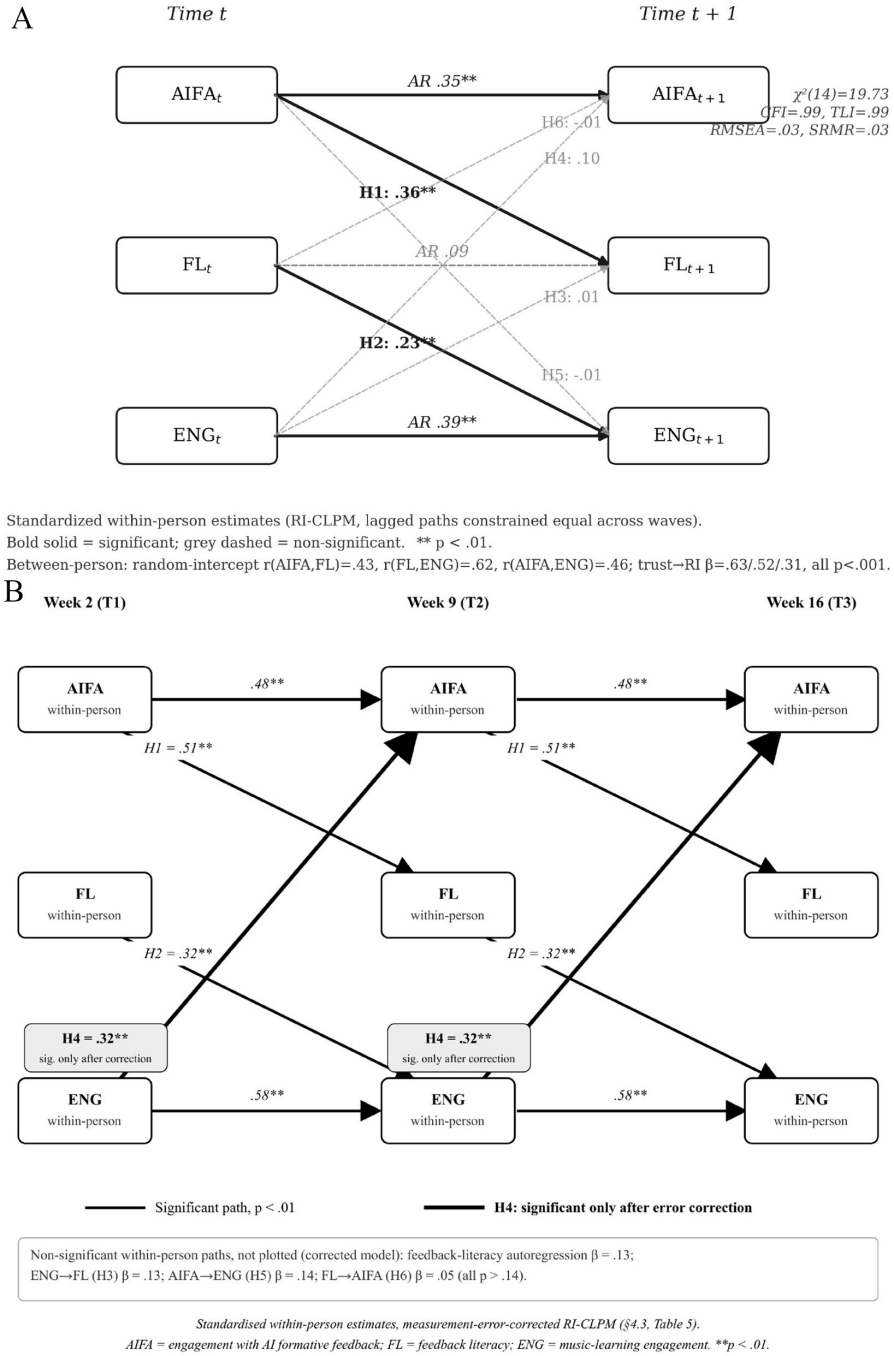
**(A)** Standardized within-person estimates from the original RI-CLPM. Bold solid paths are significant; gray dashed paths are non-significant. Table 5 reports the measurement-error-corrected estimates alongside these original values. **(B)** Plots the corrected model, with the newly significant H4 path highlighted. **p < 0.01. Standardized within-person estimates from the measurement-error-corrected RI-CLPM. Bold solid paths are significant (*p* < 0.01); the boxed H4 path (ENG → AIFA) reaches significance only after correcting for measurement error and was marginal in the original model (*p* = 0.10). Non-significant paths are not plotted; their corrected-model estimates are noted in the figure. AIFA, engagement with AI formative feedback; FL, feedback literacy; ENG, music-learning engagement.

The within-person results are summarized in Table 5 and Figure 2. Because a structural model estimated on single composite indicators carries composite unreliability into the within-person residuals (§3.4), we report both the original (manifest-composite) and the measurement-error-corrected estimates, and we treat the corrected estimates as the substantive result throughout this section.

**Table 5:** Standardized within-person estimates from the random-intercept cross-lagged panel model.

Path	Hypothesis	β (original)	*p* (original)	β (corrected)	*p* (corrected)	Result
AIFA → FL	H1	0.36^**^	< 0.001	0.51^**^	< 0.001	Supported (strengthened)
FL → ENG	H2	0.23^**^	0.001	0.32^**^	0.001	Supported (strengthened)
ENG → FL	H3 (expl.)	0.01	0.922	0.13	0.347	Not supported; equivalence inconclusive (TOST *p* = 0.077)
ENG → AIFA	H4 (expl.)	0.10	0.101	0.32^**^	0.002	Inconclusive (original) → Supported (corrected)
AIFA → ENG	H5 (comp.)	−0.01	0.822	0.14	0.143	Equivalent to negligible (TOST *p* = 0.005)
FL → AIFA	H6 (expl.)	−0.01	0.876	0.05	0.596	Equivalent to negligible (TOST *p* = 0.033)
AIFA → AIFA (AR)	—	0.35^**^	< 0.001	0.48^**^	< 0.001	—
FL → FL (AR)	—	0.09	0.385	0.13	0.305	—
ENG → ENG (AR)	—	0.39^**^	< 0.001	0.58^**^	< 0.001	—

AIFA, engagement with AI formative feedback; FL, feedback literacy; ENG, music-learning engagement; AR, autoregressive. Lagged paths constrained equal across T1 → T2 and T2 → T3 in both models. Within-person indirect effect AIFA → FL → ENG: original = 0.08, 95% Monte Carlo CI [0.03, 0.16]; corrected = 0.17, 95% CI [0.07, 0.29]. Original model fit: χ^2^(14) = 19.73, *p* = 0.14, CFI = 0.99, TLI = 0.99, RMSEA = 0.03 [0.00, 0.06], SRMR = 0.03. Corrected model fit: χ^2^(12) = 15.59, *p* = 0.21, CFI = 0.998, TLI = 0.993, RMSEA = 0.027, SRMR = 0.019. Equivalence (TOST) tests used δ = 0.15 and were run on the original model only (§4.3). ^**^p < 0.01.

Supporting H1, within-person increases in engagement with AI formative feedback predicted subsequent increases in feedback literacy, and the correction strengthened this path (original β = 0.36, *p* < 0.001, 95% CI [0.19, 0.53]; corrected β = 0.51, *p* < 0.001). Supporting H2, within-person increases in feedback literacy predicted subsequent increases in music-learning engagement, again strengthened under correction (original β = 0.23, *p* = 0.001, 95% CI [0.09, 0.38]; corrected β = 0.32, *p* = 0.001). The within-person indirect path from AI-feedback engagement to learning engagement via feedback literacy strengthened correspondingly, from 0.08 (95% Monte Carlo CI [0.03, 0.16]) to 0.17 (95% CI [0.07, 0.29]). Consistent with H5, the direct within-person path from AI-feedback engagement to learning engagement remained weak and non-significant under correction (β = 0.14, *p* = 0.14; original β = −0.01, *p* = 0.82), as expected if the relationship operates through feedback literacy rather than around it.

The reciprocal and remaining exploratory paths require a more qualified reading than in the original analysis. Within-person increases in engagement did not predict subsequent feedback literacy in either model (H3: original β = 0.01, *p* = 0.92; corrected β = 0.13, *p* = 0.35), and feedback literacy did not predict subsequent AI-feedback engagement in either model (H6: original β = −0.01, *p* = 0.88; corrected β = 0.05, *p* = 0.60). Using the original model and a smallest-effect-size-of-interest of δ = 0.15—the lower bound of the standardized effect this design was powered to detect (§3.4)—two-one-sided-tests equivalence testing supported treating H5 (TOST *p* = 0.005) and H6 (TOST *p* = 0.033) as statistically equivalent to a negligible effect; H3 showed the same pattern but did not reach conventional significance for equivalence (TOST *p* = 0.077), and we read it as consistent with, but not conclusive evidence for, a negligible effect.

H4 (engagement → AI-feedback engagement) does not fit this pattern. In the original model it was only marginal (β = 0.10, *p* = 0.10, 95% CI [−0.02, 0.21]), which we had initially read as not supported; given the width of that interval, inconclusive/underpowered is the more defensible original-model characterization. Once measurement error was corrected, however, H4 became clearly significant (β = 0.32, *p* = 0.002)—a result stable across two independent optimisation routines—indicating that within-person increases in music-learning engagement do forecast subsequent increases in engagement with the AI feedback tool. Because the corrected model's standard errors are appreciably wider than the original's (a consequence of partitioning out measurement error with only three waves), we did not additionally pursue equivalence testing on the corrected estimates for H3, H5, or H6; the substantive news from the corrected model is a detected effect (H4), not a sharper case for absence elsewhere.

Among the autoregressive paths, all three strengthened under correction: engagement with AI formative feedback (original β = 0.35, *p* < 0.001; corrected β = 0.48, *p* < 0.001), music-learning engagement (original β = 0.39, *p* < 0.001; corrected β = 0.58, *p* < 0.001), and feedback literacy, which remained the weakest and non-significant in both models (original β = 0.09, *p* = 0.39; corrected β = 0.13, *p* = 0.31). The weak autoregression for feedback literacy in particular still indicates that its within-person process carries limited temporal structure, so the cross-lagged paths running into and out of it are best read as detectable signals atop a largely fresh wave-to-wave process rather than as effects layered on strong persistence.

Taken together, correcting for measurement error—the minimum remedy available given a single composite indicator per construct per wave—does not simply sharpen the original picture; it revises it. The forward chain (H1, H2) is more strongly supported than the original analysis suggested, but the dynamic is not strictly unidirectional: engagement with music learning forecasts subsequent engagement with the AI feedback tool (H4), even though feedback literacy does not forecast AI-feedback engagement (H6) and engagement does not separately forecast feedback literacy (H3). We carry this revised, predominantly-forward-but-not-strictly-unidirectional characterization through §5.

### 4.4 Robustness and the cross-level moderation question

Results were robust to estimating the cross-lagged paths freely across transitions rather than constraining them; a complete-case vs. FIML comparison was not applicable given the absence of missing data (§4.1). As anticipated in §3.4, we did not interpret a within-person moderation of the AI-feedback-engagement → feedback-literacy path by baseline trust. A median split on trust produced a large apparent group difference in that path, but this difference is an artifact: because trust is strongly associated with the random intercepts (H7a), grouping students by trust is partly grouping them by the stable component the RI-CLPM is designed to remove, so the split-sample contrast confounds between- and within-person variance. We therefore report only the well-identified between-person role of trust (H7a) and defer the within-person moderation question to designs with more waves. As a further robustness check, we compared the original and measurement-error-corrected specifications directly (§4.3); the two agree on the forward chain (H1, H2) and on H3 and H6, but disagree on H4, which we treat as a substantive update rather than noise, given its stability across optimisation routines.

## 5 Discussion

This study asked how engagement with AI formative feedback, feedback literacy, and music-learning engagement influence one another within students over a semester, once the stable differences between students are set aside. The answer that emerges is more specific, and more forward-driven, than the reciprocal narratives common in this literature, though not as cleanly one-directional as our original analysis suggested. Within students, engaging more with AI formative feedback was followed by gains in feedback literacy, and gains in feedback literacy were followed by gains in learning engagement—both legs of this forward chain strengthened once we corrected for composite measurement error (§4.3)—and the indirect route from AI feedback to engagement through feedback literacy was discernible throughout. The specific reverse path a self-regulated-learning account emphasizes—engagement feeding back into feedback literacy—did not appear in either analysis. But a different reverse path did: once measurement error was corrected, learning engagement forecast subsequent engagement with the AI tool itself. What looks, between students, like a web of mutually reinforcing dispositions resolves, within students, into a forward chain from capability to engagement with one loop back into tool use, not into a single, clean, one-way sequence.

### 5.1 A predominantly forward, not strictly unidirectional, mechanism

The most consequential result is not, on reflection, simply a null one. Feedback literacy and engagement are routinely described as parts of a self-reinforcing loop, and at the between-person level our data are consistent with that image: students high on one tend to be high on the others, though our corrected re-estimation (§4.2) suggests this trait-level covariation is substantially carried by a shared antecedent—trust in AI feedback—rather than the three traits reinforcing one another independently. At the within-person level, feedback literacy forecast later engagement (H2), and the specific reverse path a reciprocal-loop account emphasizes—engagement forecasting later feedback literacy (H3)—did not; that particular loop ran one way in both the original and corrected models, and equivalence testing on the original model did not reach the conventional threshold for H3 (TOST *p* = 0.077); we therefore treat this path as inconclusive rather than as evidence of a negligible effect. Prior work linking feedback constructs to engagement has mostly estimated these relationships without separating stable between-person differences from within-person change, so a positive association could reflect either genuinely reciprocal dynamics or simply that engaged, feedback-literate students are consistently different people; our between/within decomposition shows both are present but at different levels, which is the more precise claim this design can support. This matters because the reciprocal framing carries practical promises—that getting students more engaged will, on its own, make them more feedback-literate—that our results do not support. Over a semester, the influence between engagement and feedback literacy appears to run predominantly from capability to engagement, not back again, echoing self-regulated-learning accounts that treat literacy-building as requiring deliberate scaffolding rather than assuming engagement alone supplies it (cf. Achuthan, 2025; on the conditions under which AI-based scaffolds do and do not translate into self-regulatory gains across cognitive, motivational, and behavioral facets).

The same one-directionality did not extend as cleanly to the AI tool itself as our original analysis implied. Feedback literacy did not forecast subsequent engagement with the AI feedback (H6), in either model, and equivalence testing on the original model supports treating this path, too, as negligible (TOST *p* = 0.033). But engagement with music learning did forecast subsequent engagement with the AI tool (H4)—a path that was only marginal before we corrected for measurement error, and clearly significant after. Two readings are possible for the paths that remained null, and the data do not decisively separate them. The reciprocal effect from engagement back into feedback literacy may be genuinely absent on this timescale: building feedback literacy may require deliberate instruction and practice that mere engagement does not supply. Alternatively, that particular reverse effect may operate over intervals shorter or longer than the roughly 7-week lag between our waves, and so be invisible here. Because the within-person autoregression for feedback literacy was itself weak (§4.3), the absent H3 path is most safely read as no evidence for reciprocity into feedback literacy on this timescale rather than as proof that the effect is zero. The H4 finding complicates any account that treats the whole system as one-directional: at minimum, students who become more absorbed in music learning also lean further into the AI tool afterward, a self-sustaining-use pattern distinct from, and additional to, the capability-building loop running through feedback literacy.

### 5.2 Feedback literacy as the conduit for AI feedback

The forward chain locates feedback literacy as the link through which AI formative feedback reaches engagement. The pattern—a significant within-person indirect path alongside a non-significant direct path (H5)—is the signature one would expect if feedback literacy is the conduit rather than a parallel correlate. We are deliberately cautious in how far we press this. Estimating within-person mediation rigorously requires more than two transitions, and with three waves the indirect effect is best read as consistent with, rather than proof of, a mediated process (Mulder and Hamaker, 2020). This within-person result speaks directly to a literature that has mostly built feedback literacy through deliberate instruction and structured tasks rather than treated it as a by-product of tool use (e.g., self- and peer-assessment interventions, structured feedback tasks); our finding that engagement with AI feedback alone forecasts within-person gains in feedback literacy suggests routine tool interaction can supply some of what those interventions are designed to build, without displacing the case for combining both. With that caveat, the result has a clear implication: the value of AI formative feedback for engagement is not automatic but conditional on students' capacity to use it. Feedback that students cannot interpret, judge, or act on has no obvious within-person route to engagement, which reframes the design problem from delivering more feedback to building the literacy that lets feedback land. This is also where our null direct path (H5) sits in useful tension with experimental work showing that AI-generated feedback can move revision behavior, motivation, and affect directly, without an explicit literacy intermediary (Meyer et al., 2024): those effects were observed on a single, immediate task, whereas ours concern durable within-person change across a semester, so the two results are compatible if AI feedback's more immediate motivational pull is not the same thing as a lasting shift in learning engagement. Similarly, work applying the Fogg Behavior Model to LLM-based tools distinguishes motivation, ability, and situational triggers as jointly necessary for sustained tool use (Jyothy et al., 2024); on that framework, our feedback-literacy conduit maps most closely onto the ability component, which leaves open—and our design cannot separately test—whether motivation and triggers operate through a different, more direct channel than the one we modeled.

This reading also speaks to a tension in the broader debate about AI in education, which often pits the efficiency of automated feedback against worries that it bypasses the student's own judgement. Our results are consistent with the two not being opposed: in this model, AI feedback was associated with engagement by way of the judgement-related capacities that constitute feedback literacy, rather than substituting for them.

### 5.3 Where AI enters: trust at the between-person level

The role of epistemic trust in AI feedback was robust but located at the between-person level. Students who trusted AI feedback more occupied higher stable positions on all three constructs, and most strongly on engagement with the AI tool itself (H7a). Trust, that is, helped explain *who* engaged with and benefited from AI feedback, even though our design could not establish whether trust shaped *how* AI feedback translated into feedback literacy from one wave to the next. This is a more modest claim than a moderation of within-person dynamics, but it is the claim the data can bear, and it keeps the distinctly algorithmic feature of the setting—students' willingness to credit a non-human judge—inside the explanation rather than outside it. This should be read as a snapshot rather than a settled disposition: work that discloses the human or AI source of feedback to students after they have evaluated it finds that this disclosure alone can shift perceived quality and trust, independent of the feedback's content (Nazaretsky et al., 2024), so the stable, trait-like reading we give trust here may itself be conditional on how transparently the tool's nature was presented to our sample over the semester. It also implies that interventions to raise trust in AI feedback (for instance, by making its basis transparent or demonstrating its accuracy on expressive as well as technical features; Berger et al., 2020) may shift students' general orientation toward the tool, while leaving open whether they accelerate the wave-to-wave process by which feedback becomes literacy.

We should be candid about a corollary. The within-person forward chain itself (H1–H2) is not, in its mechanics, specific to AI: an immediate, granular human feedback source might in principle generate the same sequence from feedback engagement to feedback literacy to learning engagement. What is distinctively algorithmic in our model lives in the trust layer—students' willingness to credit a non-human judge of qualities, such as musical expression, that they may doubt an algorithm can assess. In this study, then, the AI-specific contribution sits at the between-person level, conditioning *who* takes up AI feedback; whether algorithmic properties also reshape the *within-person* dynamics is a question this design cannot answer and that a longer panel, able to identify cross-level moderation, should pursue.

### 5.4 Implications for designing AI feedback in skill learning

Three design implications follow. First, because AI feedback reached engagement only through feedback literacy, deployments that simply increase the volume or frequency of automated feedback are unlikely to raise engagement unless they also cultivate students' capacity to use it—through, for example, scaffolds that prompt students to interpret and act on what the tool flags, rather than to receive it passively. Second, because the dynamic was predominantly forward rather than reciprocal between engagement and feedback literacy specifically, engagement-first strategies that assume a spiral into feedback literacy are misplaced here; the more reliable lever for building literacy is the feedback-literacy side of the chain, even though sustained engagement does appear to feed back into continued use of the AI tool itself (§4.3, H4). Third, because trust shaped students' general standing with the tool, attending to the credibility of AI feedback—particularly in expressive domains where students reasonably doubt an algorithm—is not a peripheral concern but part of what determines who engages at all.

### 5.5 Limitations

Several limitations bound these conclusions.

First, three waves, while sufficient to identify the RI-CLPM, are a floor rather than a comfortable margin. They constrain what can be asked: within-person mediation can be suggested but not firmly established, the autoregressive and cross-lagged structure must be assumed stationary, and a within-person moderation by trust (H7b) cannot be credibly identified. The weak autoregression for feedback literacy also cautions that, once stable differences are removed, its residual within-person process is modest, and larger samples or more waves would estimate it more precisely. Designs with four or more waves, ideally with random slopes, are needed to test reciprocity, mediation, and moderation properly.

Second, all constructs were self-reported. The three-wave separation and the method-variance checks mitigate but do not eliminate same-source concerns, and behavioral indicators of engagement with the AI tool—logged interactions, practice traces—would strengthen the case considerably. Relatedly, we measured students' engagement with AI feedback, not the quality of the feedback they went on to produce, select, or act on; work evaluating AI-supported peer feedback shows that engagement and the downstream quality of what students do with feedback are separable outcomes (Guo et al., 2024), and our design cannot speak to the latter. Third, the setting is specific: university music-education students working with a particular kind of AI formative feedback. Music was chosen because it sharpens the theoretical questions about granularity and trust, but the pattern we report should not be assumed to generalize to feedback in less quantifiable domains without test; nor should music itself be reduced to a technical-practice domain, since musical engagement also draws on attentional, affective, and self-regulatory processes that a formative-assessment frame does not fully capture (Achuthan and Khobragade, 2025). Fourth, the adapted instruments, though re-examined here, would benefit from independent validation in this population.

Fifth, non-independence from cohort recruitment is only partly resolved. Students were recruited from 22 class cohorts (*M* = 19.1 students per class) sharing instructors and a common tool deployment, which can share feedback norms, assessment schedules, and expectations about AI use; individual class and instructor identifiers were not retained in the analysis file, so we could not compute an exact cluster-robust correction, only the design-effect sensitivity bound reported in §3.4 (DEFF ≈ 1.9–2.8 depending on assumed ICC). The 22 cohorts were taught by 5 instructors; the AI tool was a required course component and usage was system-logged but not separately graded, though differences in how individual instructors framed or emphasized tool use cannot be ruled out with the data available. Standard errors here should accordingly be read as a lower bound on the true uncertainty.

Sixth, we treated engagement with AI formative feedback as a single, unidimensional construct. Although the tool's deterministic signal-processing basis (pitch, timing, and rhythm detection) is defined in §3.2, this study does not provide criterion-validity or diagnostic-accuracy evidence for those judgements, nor does it address the motivational, ability-related, and situational-trigger components that jointly determine sustained tool use in other technology-adoption frameworks (Jyothy et al., 2024). The strong association we observed between epistemic trust and the AI-feedback-engagement random intercept (β = 0.73 in the corrected model, §4.2) is consistent with trust and stable engagement not being fully distinct in students' own reports, a possibility our instruments cannot rule out. We also could not test discriminant validity between trust and perceived system accuracy specifically, because perceived accuracy of the AI tool's judgements was not separately measured in this study; only general baseline familiarity with AI practice tools was recorded (§3.2).

Seventh, assessment deadlines, recital preparation, examination periods, changes in repertoire difficulty, and access to practice facilities plausibly affected all three constructs simultaneously across the semester. The RI-CLPM separates stable person-level variance from within-person fluctuation, but it does not remove unmeasured time-varying causes of synchronous or lagged change common to a given wave; the within-person cross-lagged associations we report should accordingly be read as informative but not fully causal.

Finally, the missing-at-random assumption originally anticipated for this design was not, in the event, invoked: the realized sample had complete data at all three waves (§4.1), so questions about the robustness of within-person estimates to attrition do not arise here, though they would need to be revisited in any replication with incomplete follow-up.

### 5.6 Future directions

Beyond the present study, three extensions of the design seem most valuable. Intensive longitudinal designs—weekly diaries or experience sampling across a term—would resolve the timescale ambiguity around reciprocity and permit proper within-person mediation and moderation tests. Incorporating behavioral traces from the AI tool would complement self-report and allow engagement with AI feedback to be modeled as something students do, not only something they report. And manipulating the feedback-literacy scaffolding around the tool, rather than only measuring it, would move the forward chain from a correlational to a causal footing and test directly whether building feedback literacy is the lever our model implies. Finally, given that the corrected and original models agreed on the forward chain but disagreed on whether engagement loops back into AI-tool use, future work with more waves and a full multiple-indicator measurement model would help establish which reverse pathways are genuinely absent and which were simply attenuated by composite-level measurement error in analyses like the original one reported here.

## 6 Conclusion

When AI tools grade a student's scales, do they make the student a more engaged musician? On the evidence of this within-person analysis, the answer is a qualified yes, along a particular route, with one loop back that our original analysis missed. Engaging with AI formative feedback was followed by growth in feedback literacy, and growth in feedback literacy by growth in engagement, more strongly once we corrected for the measurement error that a single-composite structural model otherwise carries forward; the path from the tool to engagement ran through students' capacity to use what it told them, not around it. The mutually reinforcing loop that theory often assumes between engagement and feedback literacy specifically did not materialize, in either analysis. But engagement with music learning did forecast renewed engagement with the AI tool once measurement error was corrected, so the overall dynamic is better described as predominantly forward but not strictly unidirectional. Separating how students change from how they differ was essential to seeing this, and re-evaluating our own structural model once reviewers pressed on its measurement assumptions changed what we can claim about direction—both are distinctions that assessment research adopting AI tools can no longer afford to blur. The practical message is correspondingly specific: build the literacy that lets AI feedback land, attend to whether students trust it, do not expect engagement alone to close the loop into feedback literacy, but do expect sustained engagement to keep students coming back to the tool itself.

## Data Availability

The raw data supporting the conclusions of this article will be made available by the authors, without undue reservation.
